# Characteristics of diurnal ventricular premature complex variation in right ventricular outflow tract arrhythmias after catheter ablation

**DOI:** 10.1097/MD.0000000000006516

**Published:** 2017-04-14

**Authors:** Shih-Jie Jhuo, Li-Wei Lo, Shih-Lin Chang, Yenn-Jiang Lin, Fa-Po Chung, Yu-Feng Hu, Tze-Fan Chao, Ta-Chuan Tuan, Jo-Nan Liao, Chin-Yu Lin, Yao-Ting Chang, Chung-Hsing Lin, Rohit Walia, Abigail Louise D. Te, Shinya Yamada, Sunu Budhi Raharjo, Wei-Hua Tang, Kun-Tai Lee, Wen-Ter Lai, Shih-Ann Chen

**Affiliations:** a Division of Cardiology, Kaohsiung Medical University Hospital; b Division of Cardiology, Taipei Veterans General Hospital; c Institute of Clinical Medicine, and Cardiovascular Research Center, Faculty of Medicine, College of Medicine, National Yang-Ming Medical University, Taipei; d Department of Internal Medicine, Faculty of Medicine, College of Medicine, Kaohsiung Medical University, Kaohsiung, Taiwan.

**Keywords:** catheter ablation, diurnal variation, heart rate variability, recurrences, right ventricular outflow tract

## Abstract

Diurnal variations in ventricular tachyarrhythmias (VAs) have been demonstrated in idiopathic arrhythmogenic heart disease. The electrophysiological characteristics of diurnal variations in idiopathic right ventricular outflow tract (RVOT) VA have not previously been elucidated. Sixty-two consecutive patients undergoing catheter ablation for idiopathic RVOT VA (mean age: 42.8 ± 12.3 years, 35 females) were enrolled. The diurnal variation type (group 1, n = 36) was defined as those patients who had most ventricular premature contractions (VPCs) during the night hours by preprocedure Holter recordings. Group 2 (n = 26) was defined as those patients who did not have significant VPC variations. The baseline characteristics and electrophysiological properties were collected and analyzed, and the rates of recurrence after catheter ablation were compared between the 2 groups. In this study, heart rate variability analysis demonstrated lower low frequency/high frequency ratios in group 1 than in group 2 (3.95 ± 3.08 vs 6.26 ± 5.33; *P* = 0.042). There were no significant differences in baseline characteristics, echocardiography and electrophysiological characteristics between the 2 groups. During a mean follow-up period of 13.5 ± 11.0 months, a total of 16 patients had VA recurrences, including 13 patients from group 1 and 3 patients from group 2 (36.1% vs 12.5%, *P* = 0.039). This study demonstrated the effect of the autonomic nervous system in idiopathic RVOT VAs and that the diurnal variation type leads to a higher recurrence rate after catheter ablation.

## 1 Introduction

Idiopathic right ventricular outflow tract ventricular arrhythmia (RVOT VA) is one of the most common ventricular arrhythmias in the world. It is associated with structural heart disease, congenital cardiomyopathy, and ischemic heart disease. The manifestation of VAs can vary from the presence of frequent ventricular premature complexes (VPCs) to repetitive salvos, nonsustained ventricular tachycardia (VT) and incessant VTs,^[1]^ which can lead to various clinical symptoms, including palpitation, dyspnea, atypical chest pain, and syncope. The diurnal VA variant requires specialized arrhythmia management. The relationship between diurnal VA changes and heart function has been discussed in many studies.^[2,3]^ According to these investigations, the diurnal VA type affects not only heart function but also the prognosis of VA management.^[2,3]^ Factors related to diurnal changes in ventricular arrhythmia include gender, myocardial ischemia, hemodynamics, and autonomic function fluctuation.^[4,5]^ The role of the sympathetic and parasympathetic nerves in VA diurnal variations has also been extensively discussed, which may affect the use of VA medications and even play a role in VA recurrence.^[6,7]^ Catheter ablation provides another treatment option for drug-refractory VA and is highly successful, especially for cases of VA of the RVOT. However, the electrophysiological properties and ablation outcomes of diurnal variation type of idiopathic RVOT VA have not been elucidated. In this study, we aimed to investigate the characteristics and ablation outcomes in patients with diurnal type idiopathic RVOT VA.

## 2 Materials and methods

### 2.1 Patient selection

A total of 62 consecutive patients referred to the Taipei Veteran General Hospital for electrophysiological study and radiofrequency catheter ablation (RFCA) of documented drug-refractory idiopathic RVOT VAs with electrocardiographic features of typical left bundle branch block and inferior axis QRS morphology and electrophysiological study were recruited from June 2011 to December 2014. All of the clinical data were obtained after clear explanation of the study and the approval of the Institutional Review Board committee of the Taipei Veterans General Hospital (IRB No: 2015-04-002CC). Patients less than 18 years of age or those with supraventricular arrhythmia or non-RVOT VAs were excluded. Patients with multiple exits other than RVOT VAs were also excluded. Patient heart function was evaluated via echocardiography or magnetic resonance imaging before RFCA to exclude the possibility of structural heart disease. After abstaining from antiarrhythmic drugs (except amiodarone), patients with RVOT VAs were assessed by electrocardiogram (ECG) and 24-hour Holter monitoring before ablation. The total ventricular premature complex (VPC) number per 24 hours and the VPC number per hour were calculated. The VPC burden was defined as the number of VPCs divided by the total number of heart beats per hour. The VPC number and burden were also independently calculated during day-time hours (from 8 am to 8 pm) and night-time hours (from 8 pm to 8 am). According to Lambert et al's study,^[8]^ they defined that any time period that contained >35% of a patient's total VT events as the peak period of VT occurrence. We defined the diurnal variation type (group 1) as patients who had least 50% more VPCs or higher VPC burden during the night-time than VPCs or VPC burden during the day-time hours as determined by preprocedure Holter recordings.^[8,9]^ The control group (group 2) was defined as patients who did not have significant variations.

### 2.2 Heart rate variability analysis

We calculated the parameters of heart rate variability (HRV) for each enrolled patient, including SDNN, SDANN, rMSSD, pNN50, low frequency, high frequency, and the low frequency/high frequency ratio (L/H ratio). The HRV was analyzed from the Holter recordings on a Holter analysis system (Oxford Medilog Excel 2, Oxford Instrument, Abingdon, UK). The RR interval was interpreted as a premature beat if it deviated from the previous qualified interval value by more than a given tolerance (e.g., 30%), which was a programmable parameter depending on the prematurity index of ectopic beats for each patient. Abrupt temporary changes in the RR interval sequence, which represented ectopic beats, were removed, and more stationary data were acquired for the analysis with this filtering technique. Two experienced observers reviewed the RR intervals for each Holter recording. Only the RR intervals related to normal sinus beats in a stationary state were included in the analysis. In this study, reliable measurements of the HRV spectral components were achieved using this technique (<5% error) if <15% of the impulses were excluded from analysis. In calculating the HRV variables, only the normal-to-normal QRS complex intervals were included. We defined the so-called normal-to-normal beats as beats that were from the sinus impulse and not premature beats or those that were ventricular in origin.

The HRV includes time and frequency domain analyses. The time domain analysis is based on beat-to-beat or NN intervals and several parameters are calculated, including SDNN, SDANN, rMSSD, and pNN50. SDNN indicates the standard deviation of the NN intervals, which is calculated over a 24-hour period. It reflects all of the cyclic components responsible for variability during the recording period as well as total variability. SDANN represents the standard deviation of the average NN intervals calculated over short periods, and presents a measure of changes in heart rate due to cycles longer than 5 minutes. rMSSD indicates the square root of the mean of the squares of the successive differences between adjacent NNs. NN50 refers to the mean number of times an hour in which the change in successive normal sinus (NN) intervals exceeds 50 milliseconds. pNN50 represents the percentage of NN50 divided by the total NN number. In the report by Ewing et al,^[10]^ pNN50 was a reliable marker for parasympathetic activity. The pNN50 values also have a positive correlation with the high frequency in the time domain HRV analysis.

The beat-to-beat fluctuations were also transformed to the frequency domain using a fast Fourier transformation to generate the HRV spectral power. The HRV spectral power (ms^2^) was defined as the low-frequency component (LF) from 0.04 to 0.15 Hz, which indicated sympathetic activity, and the high-frequency component (HF) from 0.15 to 0.40 Hz, which represented parasympathetic activity. The low- or high-frequency components were calculated as the area under the frequency bands. The low/high frequency component ratio (L/H ratio) was calculated as an index of the autonomic balance of the heart. The mean RR cycle length was derived from the mean of all of the normal RR intervals.

### 2.3 Electrophysiological study, mapping, and radiofrequency catheter ablation

After explaining the entire procedure and obtaining informed consent, we performed a standardized electrophysiological study for all patients in a fasting state without sedation. We discontinued antiarrhythmic drugs (except amiodarone) for at least 5 half-lives before RFCA. If there was no spontaneous VA during the procedure, rapid ventricular pacing and programmed stimulation up to 3 extrastimuli were performed. If the VA could still not be induced, intravenous isoproterenol (1–5 μg/min) was infused to increase the heart rate by at least 20%, and the induction protocol was repeated. The QRS morphologies of spontaneous or induced VAs were compared with the documented VAs.

The localization of arrhythmogenic foci was performed conventionally or by using a 3D mapping system (EnsiteNavX, St. Jude, Inc., St. Paul, MN or CARTO 3, Biosense Webster, Diamond Bar, CA). We performed activation mapping and defined the earliest local electrical signals, or pace-mapping, by comparing the 12-lead QRS morphology of the paced VPCs with that of the documented VPCs. The target ablation site was selected according to the earliest activation site or the optimal pace-mapping site. Voltage mapping was also performed in the sinus rhythm. The area of endocardial bipolar voltage < 1.5 mV was defined as the low voltage zone (LVZ) and <0.5 mV was defined as the scar. The area of unipolar voltage <5.5 mV was also defined as the LVZ. We delivered radiofrequency (RF) energy by nonirrigated ablation catheter in a temperature-controlled mode at 50 to 60°C with a pulse duration of 60 seconds and a maximal power of 50 W or by irrigated catheter at 30 to 35 W to target an impedance drop of 10 ohms. Additional energy would be applied for no more than 300 seconds. Repeat mapping was performed if no VA suppression or elimination was observed. Acute procedural success was defined as complete elimination of spontaneous or inducible VAs under the infusion of isoproterenol, following the same induction protocol for 30 minutes to exclude acute recurrences. The arrhythmogenic focus was localized by the acute successful RFCA site, and the location was categorized into anterior medial, anterior free wall, posterior medial, posterior free wall of RVOT based on navigation system, or fluoroscopic images. All patients underwent a 24-hour Holter ECG monitoring after ablation.

### 2.4 Clinical follow-up

Patients were followed up in the cardiology outpatient clinic with 12-lead ECGs, 24-hour Holter monitoring, and echocardiography after RFCA every 3 months for the first year and then 6 months thereafter. Patients who could not come for outpatient follow-up in our institution were contacted by telephone for recurrent symptoms and recurrent arrhythmias. We also advised these patients to visit our affiliated institutions to complete their follow-up screenings, and we obtained medical reports from these affiliated institutions. Recurrent VA was confirmed in our hospital by medical records from the affiliated institution. Recurrence was defined as recurrence of sustained VT or nonsustained VT or greater than 1000 VPCs per day^[4]^ as confirmed by morphology criteria using 24-hour Holter ECG.

### 2.5 Statistical analysis

The data are expressed as the mean ± standard deviation for normally distributed continuous variables and proportions for categorical variables. Continuous variables were analyzed using a 2-tailed *t* test. Discrete variables were compared using a *χ*^2^ test. The Kaplan–Meier cumulative recurrence curves were plotted for predictors and the incidence of events, and survival curves were compared by the log-rank test. The associations between selected parameters and the VA recurrences after RFCA were studied by univariate Cox regression analysis. The variables selected for testing in multivariate analysis for a Cox regression model were those with a *P* < 0.05 in the univariate models. All statistical significances were set at *P* < 0.05, and all statistical analyses were carried out using SPSS 17.0 (SPSS Inc., Chicago, IL).

## 3 Results

### 3.1 Baseline characteristics of patients

**Figure 1:**
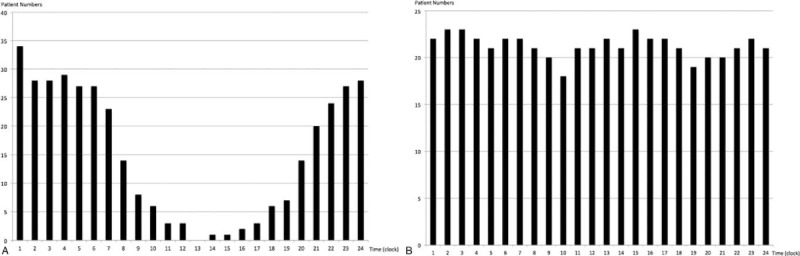
(A) The time distribution plot of VPCs in group 1 patients (diurnal type variation); (B) time distribution plot of VPCs in group 2 patients (nondiurnal type).

A total of 62 patients with RVOT VA (mean 42.79 ± 16.18 years, range 18- to 71-years old; 27 men) were enrolled in this study. The mean follow-up period was 13.5 ± 11.0 months. Thirty-six subjects were classified as group 1, and the others were classified as group 2. The characteristics of the enrolled patients are summarized in Table 1. There were no significant differences in baseline characteristics between the 2 groups. The total numbers of VPCs per day were not significantly different between the 2 groups (16378.12 ± 10615.29 vs 23269.92 ± 14891.74, *P* = 0.064). The numbers of diurnal VPCs per hour were also not different. However, the number of nondiurnal VPCs per hour was fewer for the patients of group 1 than for those in patients of group 2 (336.38 ± 320.91 vs 1000.61 ± 674.42, *P* < 0.001). The heart beats per hour during the diurnal or nondiurnal time periods were not significantly different. No significant difference was found for the VPC burden during the diurnal hours between 2 groups, but the VPC burden during the nondiurnal hours was less in the group 1 patients than in the group 2 patients (7.7 ± 6.94% vs 21.39 ± 13.59%, *P* < 0.001). The time distribution plots of the VPCs for the group 1 and group 2 patients are shown in Fig. 1A and B.

**Table 1:**
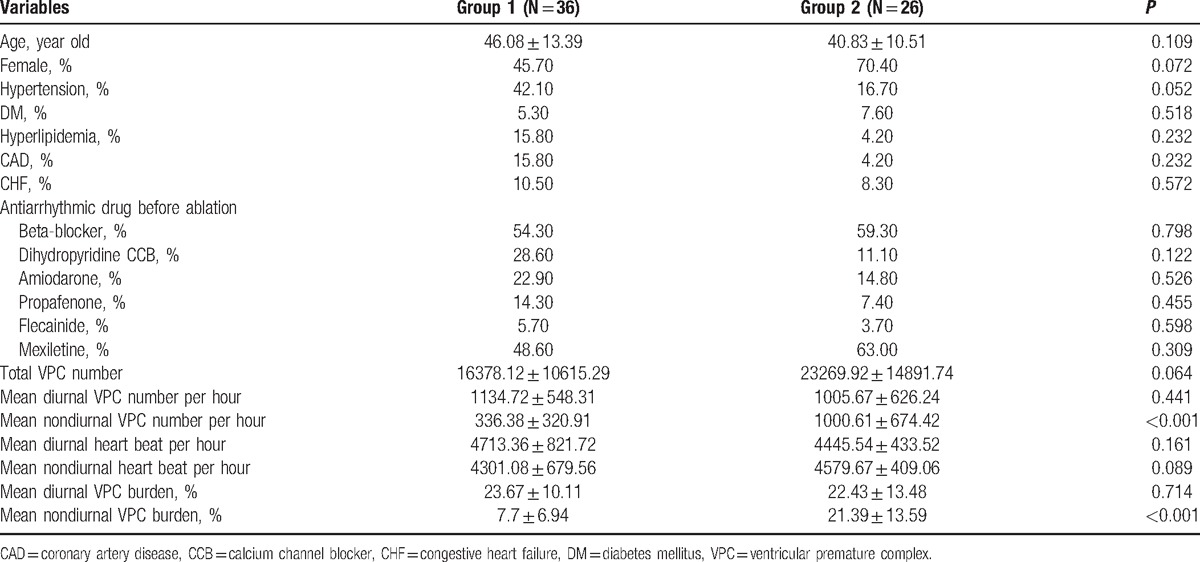
Baseline characteristics for Group 1 and Group 2 patients.

### 3.2 Heart rate variability analysis results

HRV before ablation was evaluated in each patient. In time domain analysis, there was no difference in the SDNN, SDANN, and rMSSD values between the 2 groups (Table 2). A lower pNN50 ratio was observed in the group 1 patients when compared with that of the group 2 patients (7.81 ± 11.16 vs 16.93 ± 19.42, *P* = 0.027). In the frequency domain analysis, no difference was found in the LF power or HF power (600.75 ± 890.81 ms^2^ vs 737.31 ± 1039.10 ms^2^, *P* = 0.594; 262.36 ± 405.50 ms^2^ vs 269.24 ± 431.09 ms^2^, *P* = 0.951, respectively). However, the L/H ratio was lower in group 1 than in group 2 (3.95 ± 3.08 vs 6.26 ± 5.33, *P* = 0.04).

**Table 2:**
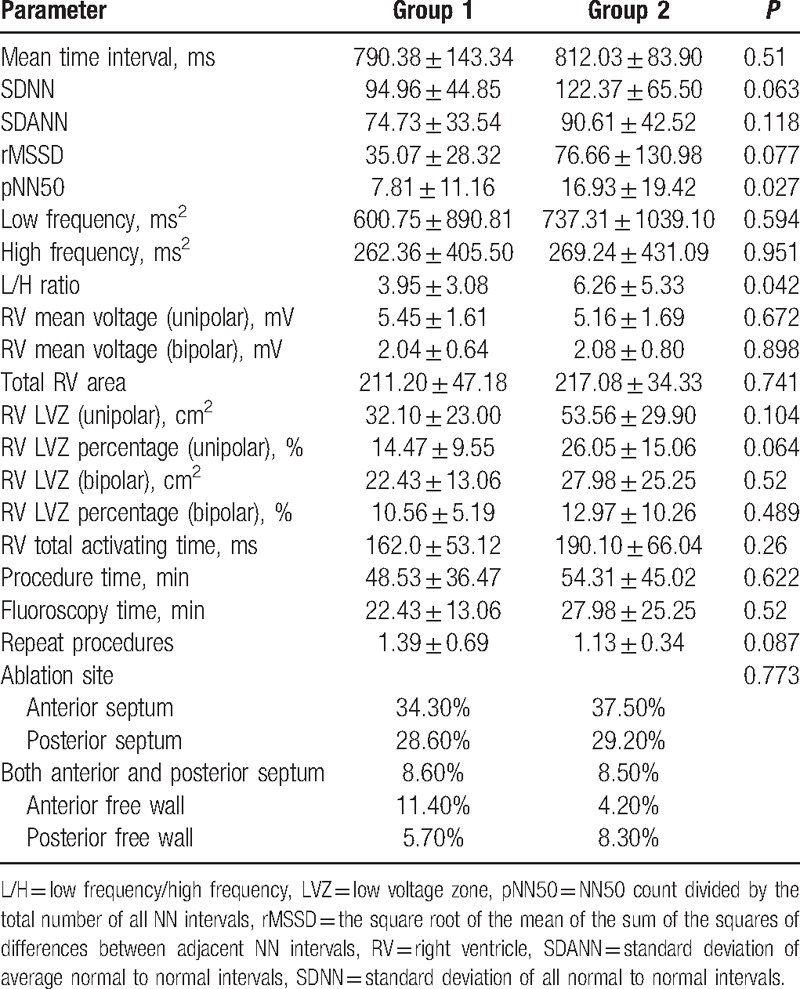
The HRV results and electrophysiological characteristics of group 1 and group 2.

### 3.3 Electrophysiologic study and ablation results

Before RVOT VA ablation, we collected electrophysiological parameters from all enrolled subjects, including mean voltage, low voltage zone area (LVZ), and activation time. The results of the electrophysiology study are listed in Table 2. There was no difference in total area and mean voltage of the right ventricular (RV) endocardium, including the unipolar and bipolar voltages. Similar areas and percentages of low voltage zones were also observed between the 2 groups. In addition, there was also no significant difference in the total RV activation time. We also compared the results and characteristics of ablation. No significant difference was found regarding procedure time, repeat procedures, fluoroscopy time, and RCFA pulses between the 2 groups. No anatomical trigger site difference for RVOT was found, including RVOT anterior septum, anterior free wall area, middle septum, middle free wall, posterior septum, and posterior free wall (*P* = 0.849). The successful RVOT ablation sites were also not significantly different (*P* = 0.773).

### 3.4 Clinical characteristics and recurrence follow-up

**Figure 2:**
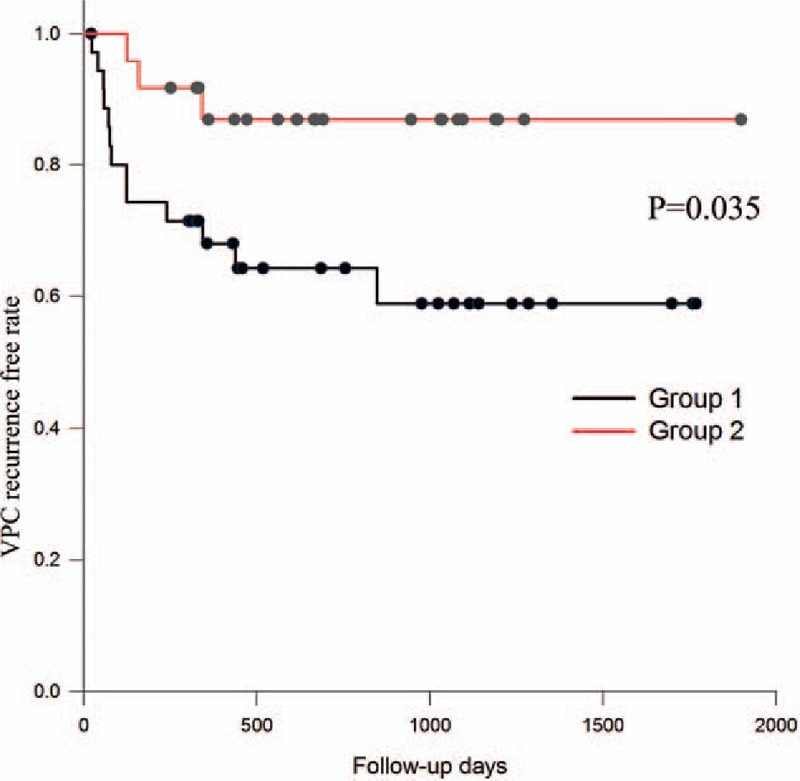
The Kaplan–Meier survival plot of recurrent VPCs (more than 1000 per day) after idiopathic RVOT ablation between group 1 and group 2.

**Figure 3:**
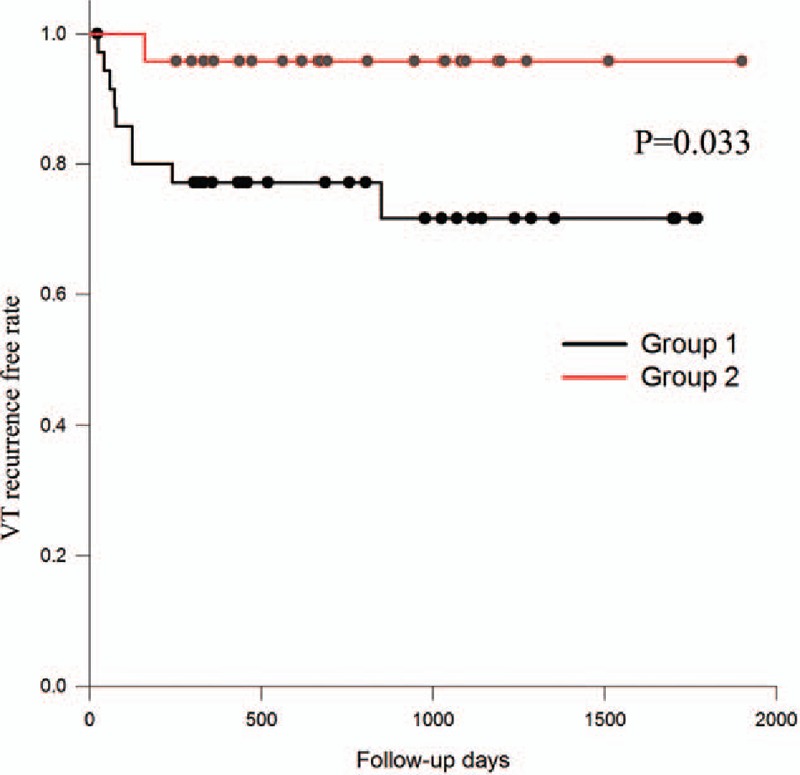
The Kaplan–Meier survival plot of recurrent VT after idiopathic RVOT ablation between groups 1 and 2.

After ablation, we followed the clinical characteristics and recurrence of enrolled patients. After a follow-up period of 13.5 ± 11.0 months, the echocardiography revealed there were no differences in the functional parameters of the left ventricle, including systolic, diameter, diastolic diameter, and ejection fraction (Table 3). For the right ventricle, there was also no significant difference in ejection fraction and systolic pressure. However, the mean days of VPC recurrence for the group 1 patients was significantly shorter than that for patients in group 2 (1140.42 ± 137.10 vs 1677.51 ± 119.47, *P* = 0.035) (Table 3). The mean recurrence days of VT were 1334.82 ± 124.23 in group 1 and 1826.54 ± 70.93 in group 2 (*P* = 0.033) (Table 3). The Kaplan–Meier plots are illustrated in Figs. 2 and 3. The univariate and multivariate Cox regression analyses elucidated that diurnal type VPC was independently associated with VPC recurrence (hazard ratio [HR]: 3.535; 95% confidence interval [95% CI]: 1.006–3.94; *P* = 0.049; HR: 3.524; 95% CI: 1.003–12.379; *P* = 0.049) (Table 4). At the same time, it was also an independent factor for VT recurrence (HR: 8.463; 95% CI: 1.092–65.58; *P* = 0.041; HR: 8.495; 95% CI: 1.096–65.826; *P* = 0.041) (Table 4).

**Table 3:**
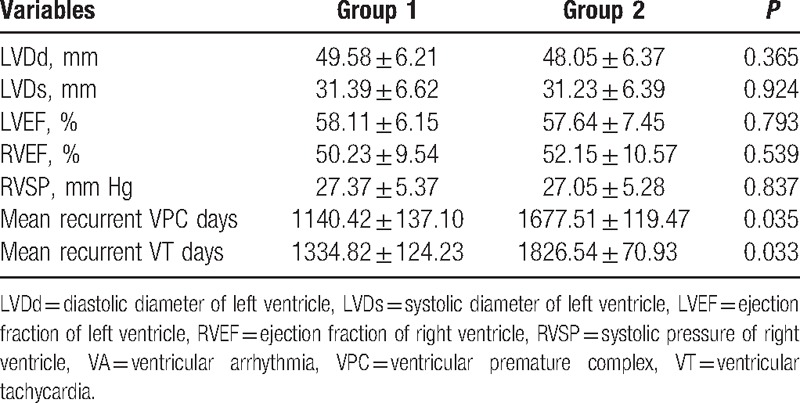
The follow-up characteristics of echocardiography and recurrence days of VA.

**Table 4:**
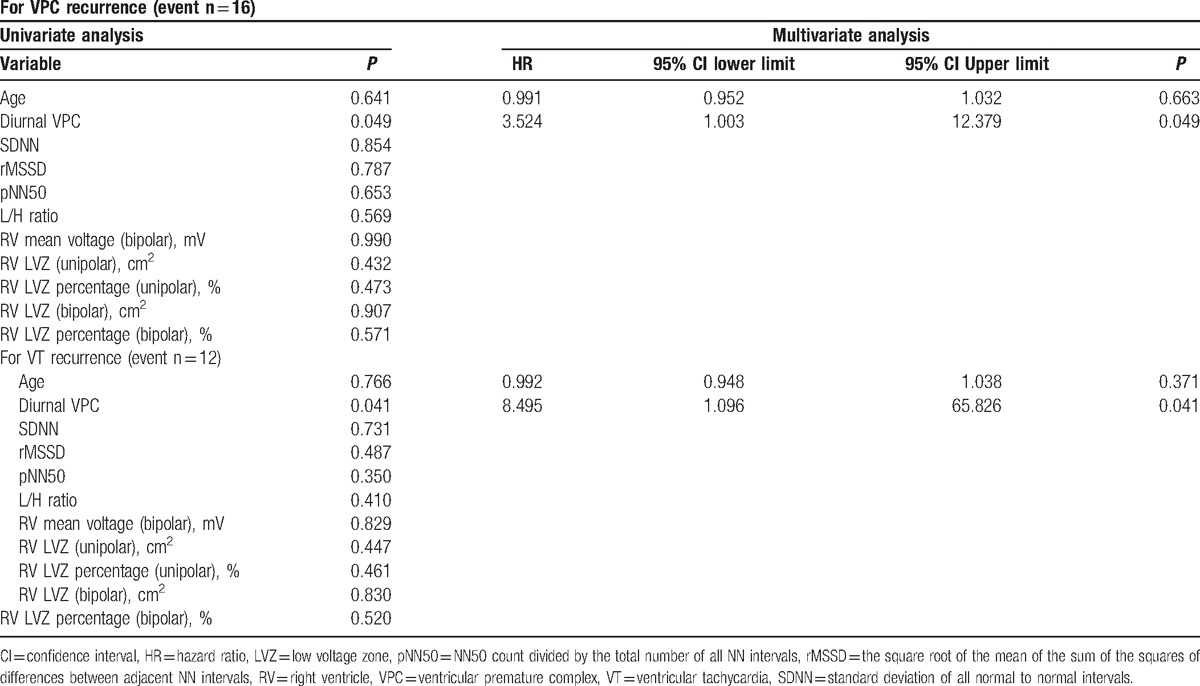
Cox regression analysis for VPC and VT recurrence.

## 4 Discussion

In this study, we found that the RV substrate properties (both unipolar and bipolar voltages) were similar between patients with or without diurnal VPC variations. In addition, there was no difference in the RVOT trigger location in both groups. These data suggest that the autonomic inputs only affected the VPC/VT attack time without electrical or structural remodeling to the RV and RVOT; therefore, it was reasonable for us to apply a similar ablation approach to these 2 types of RVOT-VA. However, group 1 patients demonstrated poor outcomes after catheter ablation with high recurrence rate, suggesting poor responses to catheter ablation in patients with diurnal-type RVOT-VA. Careful follow-up for recurrence in this patient population is recommended.

The diurnal characteristics of ventricular arrhythmias have been extensively studied.^[2,11]^ Different types of diurnal variations result in different prognoses. Recently, catheter ablation has become an important treatment option for drug-refractory ventricular arrhythmia in clinical practice; however, the impact and influence of diurnal characteristics for ventricular arrhythmia ablation remain unclear and related studies are rare. In our study, we presented the relationship between diurnal VPC variations and ablation outcomes for RVOT VAs. Although the economic factors, limited patient numbers, and number of repeated ablation procedures (due to the lessen severity of VA symptoms after the first ablation) were similar between the 2 groups, patients with diurnal type VPCs had fewer arrhythmia recurrence-free days, including VPC and VT recurrence days. The diurnal VPC variation can be associated with heart function, heart rate, and clinical prognosis. According to a study by Gillis et al,^[12]^ for patients with left ventricle ejection fractions (LVEF) > 0.30, a distinct VPC variation and an expected increase in morning VPC frequency were presented. In a study by Guo and Stein,^[13]^ similar results revealed that the diurnal features of cardiac rhythm determined cardiovascular risk and clinical outcomes, especially the morning rhythm. For patients with significant diurnal variations, they usually preserved β-adrenoceptor, autonomic function or catecholamine stores in the heart and are responsive to sympathetic stimulation, which may result in ventricular arrhythmia.^[14]^ Recently, some studies have revealed that the nighttime variations in cardiac rhythm also influence the clinical outcomes in patients with sudden cardiac death and contributes to malignant ventricular arrhythmia.^[9,15]^

Previous studies have revealed that autonomic function plays an important role in cardiac arrhythmia.^[16]^ Generally, sympathetic nerve activation was believed to trigger ventricular arrhythmia. Sympathetic hyperactivity promoted the occurrence of VT. For patients with diurnal rhythms, they may have preserved autonomic function, including sympathetic or vagal activity.^[14]^ Preserved vagal activity was augmented in response to the sympathetic hyperactivity and had a protective effect for ventricular arrhythmia. However, in patients with vagal withdrawal who had diurnal rhythms, vagal tone was reduced after sympathetic hyperactivity, which triggered ventricular arrhythmia.^[14,15]^ Similar results were elucidated in our study. The group 1 patients had similar VPCs and VPC burden during the diurnal period but significantly different values during the nondiurnal period compared with group 2 patients. These data implied that group 1 patients had higher vagal activity during the nondiurnal period, which resulted in fewer VPCs. Higher VPCs during the diurnal period might be related to vagal withdraw and enhanced sympathetic effect. In addition, patients with diurnal VPCs tended to have lower rMSSD and pNN50 values. These observations implied that vagal tone withdrawal triggers VA and results in fewer recurrence-free days of RVOT VAs after ablation.

Compatible with our HRV analysis, the effect of the autonomic system on the idiopathic RVOT-VA substrate was not significant. Unlike the autonomic effect on atrium arrhythmia reported by Lo et al,^[17]^ we could not find significant differences in ventricular substrate electrophysiological mapping, including mean voltage, LVZ area, and activation time. According to a report by Huang et al,^[18]^ the effect of sympathetic activity on the endocardium substrate was significant mostly in patients with organic heart disease. However, in our study, most enrolled patients had idiopathic RVOT-VA and did not have significant structural heart disease. This may explain why there was no significant autonomic effect on the ventricular substrate in our investigation.

In our study, there were some limitations. Our study number was limited, and no significant difference in sympathetic activity or vagal activity was elucidated following HRV analysis. Although several parameters such as rMSSD correlated to vagal activity and tended to be lower, they were still not statistically different. According to the report of Buchheit and Gindre,^[19]^ pNN50 has better correlation to cardiorespiratory fitness. rMSSD might be influenced by confounding factors and did have statistically difference but pNN50 revealed significant difference. More study patients might demonstrate characteristics of diurnal VPCs in the frequency domain analysis. However, the ratio of low frequency and high frequency was different between the 2 groups. One possible explanation was that short-term HRV analysis could be influenced by gender and age.^[20]^ Differences in age and gender could mask or influence the HRV results. In our study, although there was no significantly different distribution of gender between the 2 groups, the age distribution of the enrolled patients was ranged from 18 to 71 years, which may have confounded our HRV results.^[20]^ Therefore, the L/H ratio is much more robust in excluding the age effect on LF and HF. Another possible reason was that VA may be triggered by variations in the autonomic function balance in patients with circadian rhythms and preserved autonomic function. For these patients, individual sympathetic or vagal activity alone might be insufficient to determine the VA trigger factors. Similar results have also been demonstrated in animal studies and revealed the importance of cardiac autonomic balance in preventing lethal arrhythmia.^[21]^ In addition, we did not routinely use isoproterenol to induce VAs for every study patient, especially for patients with frequent spontaneous VPCs in our study. Thus, we could not obtain enough information regarding the response of VPCs or VT under isoproterenol infusion during ablation, which might present autonomic characteristics between 2 groups. We also did not observe vagal effects during ablation. Because there is not GP like structure in the epicardial side of ventricular myocardium, most of the major autonomic innervation coming from the root of great vessel (ex: ascending aorta), and ventricular myocardium is also thicker than atrial myocardium. When performed endocardial ablation, it may not affect the epicardium significantly. Therefore, no obvious vagal effect revealed during RFCA in our study.

Recently, RFCA for RVOT-VA has provided another important treatment option and has become a growing trend in clinical practice. Understanding the possible factors that impact prognosis and recurrence for RVOT-VA ablation is critical. Our study highlighted the importance of VPC characteristics for RVOT-VA and its impact on ablation prognosis. The influence of the autonomic system seems insignificant for the ventricular substrate in idiopathic RVOT-VA. However, the diurnal variation ventricular rhythm type may be a major prognostic factor for RVOT-VA ablation.

## 5 Conclusion

This study demonstrates that the autonomic input for RV and RVOT causes the diurnal variation only in the VPC/VT attack, without electrical and structural remodeling. There was no difference in vagal or sympathetic activity alone to result in a different trigger location for RVOT VAs. However, patients with diurnal VPC variations demonstrated poor outcomes after catheter ablation with higher recurrence rates, suggesting poor responses to catheter ablation in patients with diurnal variation type RVOT-VA. Careful follow-up for recurrence in this patient population is recommended.
